# Machine learning to promote translational research: predicting patent and clinical trial inclusion in dementia research

**DOI:** 10.1093/braincomms/fcae230

**Published:** 2024-07-25

**Authors:** Matilda Beinat, Julian Beinat, Mohammed Shoaib, Jorge Gomez Magenti

**Affiliations:** Institute of Psychiatry Psychology and Neuroscience, King’s College London, SE5 8AB, London, UK; Independent Researcher, 1071XA, Amsterdam, The Netherlands; School of Life and Medical Sciences, University of Hertfordshire, AL10 9AB, Hatfield, UK; Magenti Methodologies, CB4 3JG, Cambridge, UK

**Keywords:** Alzheimer’s disease, machine learning, patents, clinical trials, classification

## Abstract

Projected to impact 1.6 million people in the UK by 2040 and costing £25 billion annually, dementia presents a growing challenge to society. This study, a pioneering effort to predict the translational potential of dementia research using machine learning, hopes to address the slow translation of fundamental discoveries into practical applications despite dementia’s significant societal and economic impact. We used the Dimensions database to extract data from 43 091 UK dementia research publications between the years 1990 and 2023, specifically metadata (authors, publication year, etc.), concepts mentioned in the paper and the paper abstract. To prepare the data for machine learning, we applied methods such as one-hot encoding and word embeddings. We trained a CatBoost Classifier to predict whether a publication will be cited in a future patent or clinical trial. We trained several model variations. The model combining metadata, concept and abstract embeddings yielded the highest performance: for patent predictions, an area under the receiver operating characteristic curve of 0.84 and 77.17% accuracy; for clinical trial predictions, an area under the receiver operating characteristic curve of 0.81 and 75.11% accuracy. The results demonstrate that integrating machine learning within current research methodologies can uncover overlooked publications, expediting the identification of promising research and potentially transforming dementia research by predicting real-world impact and guiding translational strategies.

## Teaser

Beinat *et al*. examined the growing challenges of dementia, projected to impact 1.6 million in the UK by 2040 and incur annual costs of £25 billion. Their study uses machine learning to predict the translational potential of dementia research, increasing the discoveries’ turnover rate by analysing 43 091 UK publications.

## Graphical Abstract

**Graphical Abstract:**
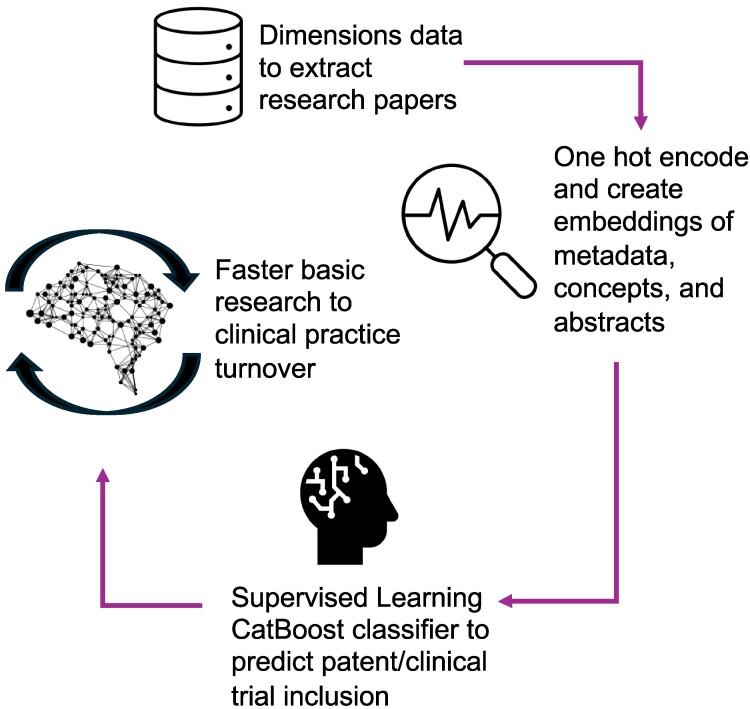

## Introduction

Almost 1 million people live with dementia in the UK, with a forecasted 1.6 million people to be affected by 2040. The increasing incidence rate in the 2010s marks a concerning trend reversal compared with other Western nations. The economic burden of dementia-related diseases is substantial, costing UK society ∼£25 billion annually.^1^

At the same time, the last decade has seen a notable surge in dementia research publications from UK institutions, attributed to increased government investment since the mid-2010s and the rise of organizations like Alzheimer’s Research UK. In 2022 alone, over 3000 dementia research papers were published in the UK.^2^

This burgeoning research landscape reveals a significant gap between scientific advancements and tangible health outcomes, underscoring the urgency to expedite and facilitate the translation of fundamental research into real-world benefits for patients with dementia and their families. The vast data processing capabilities of machine learning (ML), analysing extensive data pools within seconds, positions it as a promising potential tool to improve decision-making when addressing this translational challenge.^3^

### The slow pace of progress

The amyloid hypothesis was postulated more than 30 years ago.^4^ Only in the past 2 years, the first two drugs targeting beta-amyloid accumulation in the brain demonstrated clinical efficacy in people with early-onset Alzheimer’s disease. This contrasts with the cumulative private expenditure on clinical trials for Alzheimer’s disease, estimated to be $42.5 billion over a 25-year span. This estimate does not include the cost of the research leading up to those Phase 1 trials, which is also notorious for being a lengthy and complicated process: the beginning of the ‘valley of death’ in drug discovery.

This is not a problem unique to dementia research. In fact, most biomedical research does not translate into meaningful benefits for the people whom the research is trying to serve, and when it does, it often takes more than two decades to come to fruition.

Explanations about the slow pace of progress in dementia research and neuroscience more generally tend to focus on factors such as the complexity of the human brain, the challenges with reproducibility of preclinical data or the lower investment in the field of neuroscience compared with other disease areas with similar economic costs to society.^5^ This manuscript highlights a fundamental issue: identifying innovative ideas is challenging, necessitating new tools to assess research’s translational potential. ML, capable of analysing large data sets and identifying trends, can significantly aid this evaluation.

### Peer review and innovation

Peer-review processes, crucial in funding and publication decisions, often consider scientific quality and potential impact. However, it has been found to be an inconsistent process, and the literature is rich with examples of disparities derived from the peer-review process, related to gender, ethnicity, research institution and geographic location.^6^ Two recent examples in the field of dementia research highlighted that the current review model that informs which ideas receive funding and the type of research that is published favours particular researchers over others. This is a considerably well-established phenomenon, to the point that lottery systems to award research funding have started being introduced by funding organizations.

Frameworks to foster translational impact, such as the Framework to Assess the Impact from Translational health research (FAIT), have been developed, but they primarily focus on *post hoc* evaluation of research impact. Such frameworks, while valuable, do not inherently expedite the process of translating discoveries into tangible benefits for patients.

### Novel methodologies

Recent innovations have improved translational research identification and evaluation. For example, Manjunath *et al*.^7^ used patent citations to map research articles, revealing differences in text, meaning and author demographics, highlighting biomedical impact. Similarly, Nelson *et al*.^8^ delved into 43.3 million papers, employing deep learning models that amalgamate metadata and abstract text. They demonstrated that advanced models outperform traditional citation metrics in predicting a paper’s impact on patents, guidelines and policies. Cao *et al*.^9^ used text mining and predictive modelling to study how scientific concepts’ characteristics and network positions aid their application transition. More recently, Li *et al*.^10^ introduced a novel Translational Progression (TP) measure, rooted in biomedical knowledge representation. Analysing over 30 million PubMed articles, their method traces biomedical research’s translational path, suggesting that it could help policymakers monitor high-potential research in real time.

Collectively, these methodologies display a significant shift in the landscape of research tools and models. They can prove instrumental in bridging the gap between scientific discovery and practical healthcare applications, a particularly poignant challenge in the field of dementia research.

### Contribution to the field

In this work, we developed an ML model capable of scanning research paper metadata—such as author names, research organizations, and so on—and giving a prediction on whether that paper will be cited by patents or clinical trials. This tool can facilitate the process of identifying fundamental research with the highest ‘translational potential’.

Alongside paper metadata, we also investigated the impact of content-based features on the model’s predictive analysis. A model that can help understand the translational potential of research based on the scientific content of publications could have significant implications in the decision-making processes of funders, research organizations or scientific journals.

To the best of our knowledge, this is the first analysis in the dementia research field, using a novel ML framework, with the potential to predict the translation of fundamental research.

## Materials and methods

This paper uses bibliometric data available through the Dimensions API (https://www.dimensions.ai/products/all-products/dimensions-api/). The code used to extract data from Dimensions, the clean data to create the ML model and the Python code (version 3.12) created to train the CatBoost ML model are available at: https://github.com/MatildaBeinat/ML-for-Translational-Research. Additional information can be found upon contact with the authors.

### Data

The data set was downloaded from Dimensions, a scientific database that includes data on 141 million publications, 158 million patents, 7 million grants and 811 000 clinical trials (as of January 2024). Dimensions consists of a comprehensive list of publications linked via citations allowing for a succinct view of the landscape of today’s research, mapping the research cycle from input to output.^2^

Metadata on 43 091 publications in the field of dementia research was extracted with python code, using Google Colab as our IDE. These publications were produced by institutions based in the UK between 1990 and 2023. We extracted and analysed data using Dimensions Search Language and Python 3.12, organizing it for the ML model on Google Colaboratory. GPT-4, through the chatGPT interface, assisted in optimizing and refining the code. The full feature list is summarized in Supplementary Table 1, along with their definition in Supplementary Table 2.

Time-sensitive metrics such as ‘recent citations’, ‘altmetric score’ and ‘times cited’ were excluded from the model to avoid label leakage. We only included Dimensions-provided concepts with high-importance scores to keep the ML model relevant and omitted concepts appearing fewer than 20 times to prevent clutter. Abstract embeddings were used, excluding titles to avoid duplication, to avoid unnecessary word embedding duplication. We processed abstracts with OpenAI’s ADA-02 text embedding tool, truncating longer abstracts due to tool limitations and using parallel processing for efficiency.

When all features in Supplementary Table 1 were included in the training set, the model performed best, as evidenced by the model metrics.

Patent and clinical trial data for each publication were accessed and added. These particular data were used to create the label for the ML model.

Data including concepts, research activity codes and disease categorizations were one-hot encoded and formatted as lists. Feature reduction for these, plus abstracts, used truncated singular value decomposition.

### Machine learning model

We selected CatBoostClassifier, among the many ML tools available, which is a modern and popular ML classifier used for its high accuracy and ability to handle numerical and categorical data simultaneously. We used Google Colab as our Python IDE to clean and prepare data, train the model and review the results. We used Google Colab forms for a user-friendly interface, allowing for a simple way to adjust and alter parameters.

The label was created based on whether the publication was cited in a patent or clinical trial, allowing the model to predict a publications inclusion/exclusion from patent/trial. With a classification model, the label required to be binary: 0 or 1. We chose to label a publication with ‘0’ if no prior citations within a patent or clinical trial were found in the database, and ‘1’ for publications with a minimum of one citation in a patent or clinical trial. For the purposes of this work, no difference was made in the classification between papers with a single patent citation and papers with multiple patent citations.

We split the data between 75 and 25 for training and testing, respectively. We optimized the model for accuracy; however, the user can choose the performance metric for the model, selecting from Accuracy, Precision, Recall or F1 Score. Furthermore, the user has the option to select the specific label for training the model, either ‘Patents’ (label_patents) or ‘Clinical Trials’ (label_trials).

We rebalanced our data set to address the skew towards publications lacking citations in patent or clinical trials, down-sampling the majority class by 85% for patents and 95% for clinical trials. This rebalancing prevents the model from overfitting to the majority class. We avoided oversampling the minority class due to its poor performance in large data sets. The down-sampling achieved a 1:1.04 (2647 ones, 2733 zeros) balance for patents and 1:1.54 (660 ones, 1011 zeros) for clinical trials (Supplementary Fig. 1). The rebalanced data set was held throughout training and testing of the model.

### Models per label

For each label, label patents and label clinical trials, three models were trained to examine the effect of different feature sets. Model 1, the simplest one for each of the two labels, was trained using publications’ metadata only. This model provides a baseline to test the model’s performance when including content-based information in the feature set. For both prediction problems, Model 2 was trained using metadata alongside concept embeddings. Finally, Model 3 was trained with metadata, concept and abstract embeddings. The final model consisted of the most elaborate thematic data, allowing for the model to learn which characteristics of a publication, including its scientific content extracted from embeddings, led to patent or clinical trial inclusion or exclusion.

The performance for each model was measured by the accuracy, lift score, area under the curve (AUC) and precision–recall curves. We defined the lift score as the accuracy of the model minus the prevalence of zeros in the label input, a way to measure the effectiveness of the predictive model. AUC scores were visualized in an receiver operating characteristic (ROC) curve, which measures the classifiers ability to distinguish between the labels. We also used precision–recall curves, which are more resistant to the bias of imbalanced data sets.

We further analysed the model’s capability to predict accurately throughout time by inferring the label of 10 randomly selected publications for each year within our data set, with equally split positive and negative labels. We plotted the Δ label (real label − predicted label) for each publication with a threshold of 0.5 and −0.5 to visualize which publication was labelled inaccurately by the model.

## Results

### Distribution of publications

Over the period of January 1990 to October 2023, 43 091 dementia research papers were published in the UK. Of these research papers, 3026 research papers were cited in a patent and 850 research papers were cited in a clinical trial. This timeframe was selected in order to obtain a sufficiently large number of publications for analysis and to allow enough time for citations from patents and clinical trials to develop. Of these publications, 36 850 were suitable for analysis; the rest had insufficient data in the feature list and were excluded. Taking into account, papers published up until 2017, 20 864 papers were used to train and test the model. This selection was made during the data preparation phase (Supplementary Fig. 1).

Upon exploring our data set, we found that research papers that were cited in a patent were on average cited by 245.93 other papers, while research papers that were not cited in a patent were on average cited by 30.85 other papers. A two-sample unequal variance *t*-test confirmed the significant difference in citations (*P* < 0.001).

Alongside that, research papers that were cited in clinical trials were on average cited by 382.24 other papers, while papers that were not cited in clinical trials were on average cited by 39.19 other papers. A two-sample unequal variance *t*-test confirmed the significant difference on citations (*P* < 0.001).

The average time delay from paper publication to patent citation was 4.28 years (Supplementary Fig. 2). The average time delay from publication to clinical trial citation was 6.57 years (Supplementary Fig. 3), showing that it takes longer for fundamental research to influence clinical trials than patents. For the training and testing of our model, we chose to exclude papers published after the year 2017, to allow enough time for the papers to have been cited in a patent or clinical trial.

### Model performance

#### Label patents

**Figure 1:**
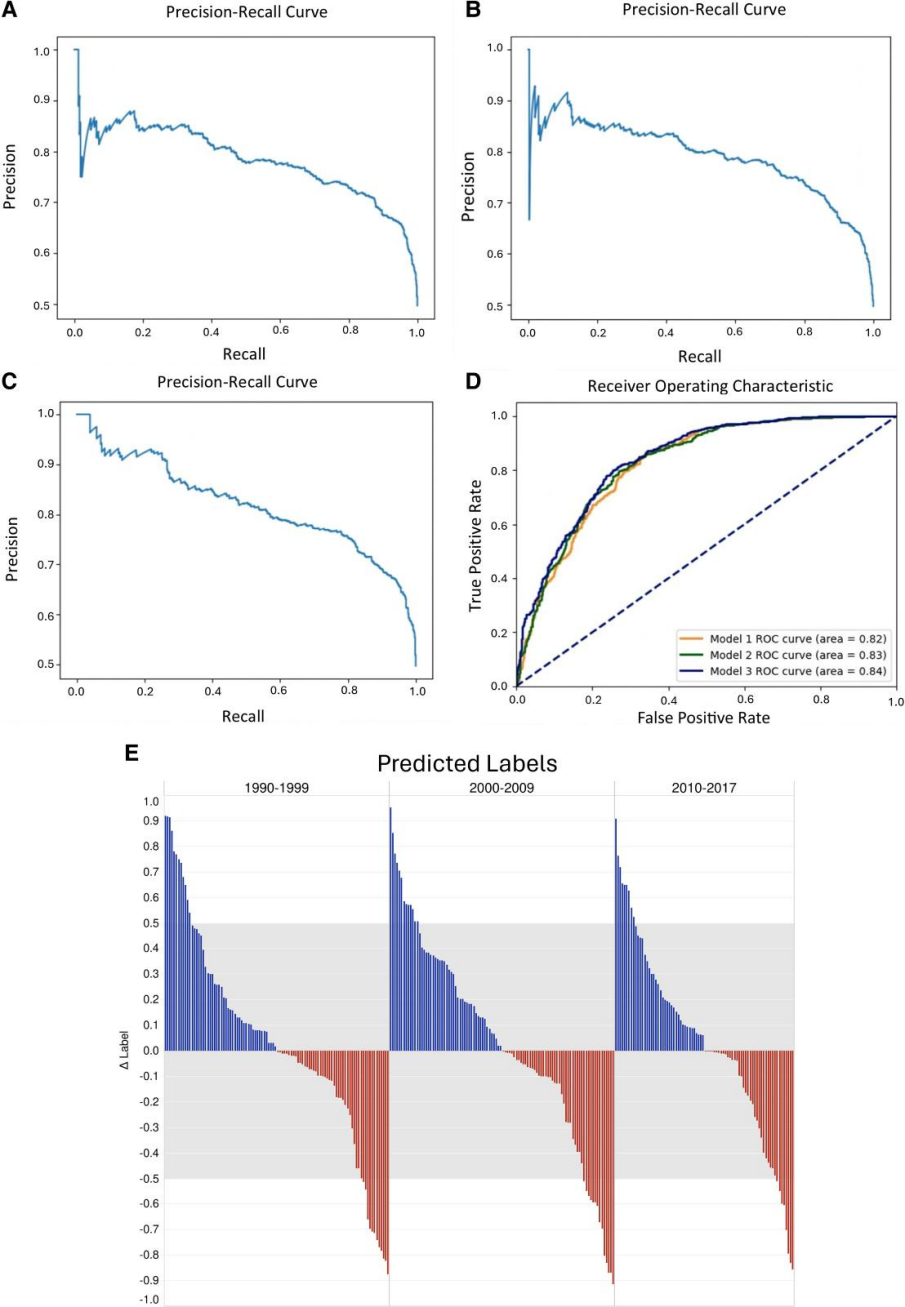
**Precision–recall curves and area under the receiver operating characteristic curve (AUROC) for label patents.** (**A–C**) Precision–recall curves for Models 1, 2 and 3, respectively. Model 1 trained and tested on only metadata, Model 2 trained on metadata and concept embeddings and Model 3 trained on the aforementioned including abstract embeddings for the prediction of label patents. The models are plotted against their respective precision and recall outputs. The yellow ROC curve (**D**) depicts Model 1, green is for Model 2 and blue is for Model 3. (**E**) Delta (Δ) label is plotted for 10 publications per year for the time frame 1990–2017. The grey box depicts the threshold that decides whether the publication will be cited in a patent or not. The predicted labels within the grey box are the correct predictions, and all other publications have been mislabelled by the model.

Model 1 returned an AUC of 0.82, with an accuracy of 75.53%, precision of 73.42%, recall of 79.67%, F1 of 74.42% and lift score of 25.28%. The full model metrics alongside the confusion matrix are shown in Table 1. The precision–recall curve is shown in Fig. 1A.

**Table 1:** Model 1: Metadata model label patents metric values and confusion matrix

Metric	Values (%)		Confusion matrix	
Accuracy	75.53		Predicted (0)	Predicted (1)
Precision	73.42	True (0)	483	193
Recall	79.67			
F1	76.42	True (1)	136	533
Lift	25.28			

In contrast, the combination of metadata and concept embeddings as feature input (Model 2) returned an AUC score of 0.83, an accuracy score of 76.28%, precision of 74.44%, recall of 79.67%, F1 of 76.97% and a lift score of 26.02%. Model 2, trained on both metadata and concepts, outperforms Model 1, which was trained just with metadata as feature input. A full visualization of the model metrics is summarized in Table 2. The precision–recall curve is shown in Fig. 1B.

**Table 2:** Model 2: Metadata and concepts model label patents metric values and confusion matrix

Metric	Values (%)		Confusion matrix	
Accuracy	76.28		Predicted (0)	Predicted (1)
Precision	74.44	True (0)	493	183
Recall	79.67			
F1	76.97	True (1)	136	533
Lift	26.02			

The most complex model for patent prediction was trained on metadata, concept embeddings and abstract embeddings as feature input (Model 3). This model resulted in the best model of the three trained and tested, returning an AUC score of 0.84, an accuracy value of 77.17%, precision, recall and F1 of 75.21, 80.72 and 77.87%, respectively, with a lift score of 26.91%. The full metrics are summarized in Table 3, and precision and recall curve is shown in Fig. 1C.

**Table 3:** Model 3: Metadata, concepts and abstracts model label patents metric values and confusion matrix

Metric	Values (%)		Confusion matrix	
Accuracy	77.17		Predicted (0)	Predicted (1)
Precision	75.21	True (0)	498	178
Recall	80.72			
F1	77.87	True (1)	129	540
Lift	26.91			

We plotted the three models’ AUCs onto one ROC curve for a better visualization of their predictive performance. As seen in Fig. 1D, the model that outperforms the rest is Model 3 with standard metadata, concepts and abstract embeddings as feature input, while the model with only standard metadata as feature input underperforms each model in label trials.

The data we are analysing are highly time sensitive. This stems from the inherent nature of publications, which require time to be noticed, cited and to contribute value to the field. Consequently, the label might be affected by time-dependent factors, for which we do not have explicit controls in the models. To ratify this, we removed highly time-dependent features (e.g. recent citations, times cited and altmetric scores) from the models, to prevent their influence. We assessed our models’ time sensitivity by testing their consistent label prediction over time with our test data set. We explored this by randomly selecting five publications with Labels 0 and 5 with Label 1 for every year within our timeframe 1990–2017, analysing 270 publications in total. We compared the real label of each publication with the predicted label calculated with Model 3 for label patents (metadata, concepts and abstract embeddings as features). The predicted label in this case is the per cent chance the model expects the publication to be cited in a patent: below 50% the model will assign a Label 0, above 50% the model assigns a Label 1.

To determine the difference between the predicted label and the real label, we plotted the Δ label for each individual publication. The closer the Δ label value is to 0, the better the model performed (Fig. 1E).

We calculated the predictive values to depict the degree of inaccuracy of the model. We found that the model performs similarly through time (74% accuracy in the 1990s, 73% accuracy in the 2000s and 79% accuracy in the 2010s; Supplementary Table 3).

#### Label trials

**Figure 2:**
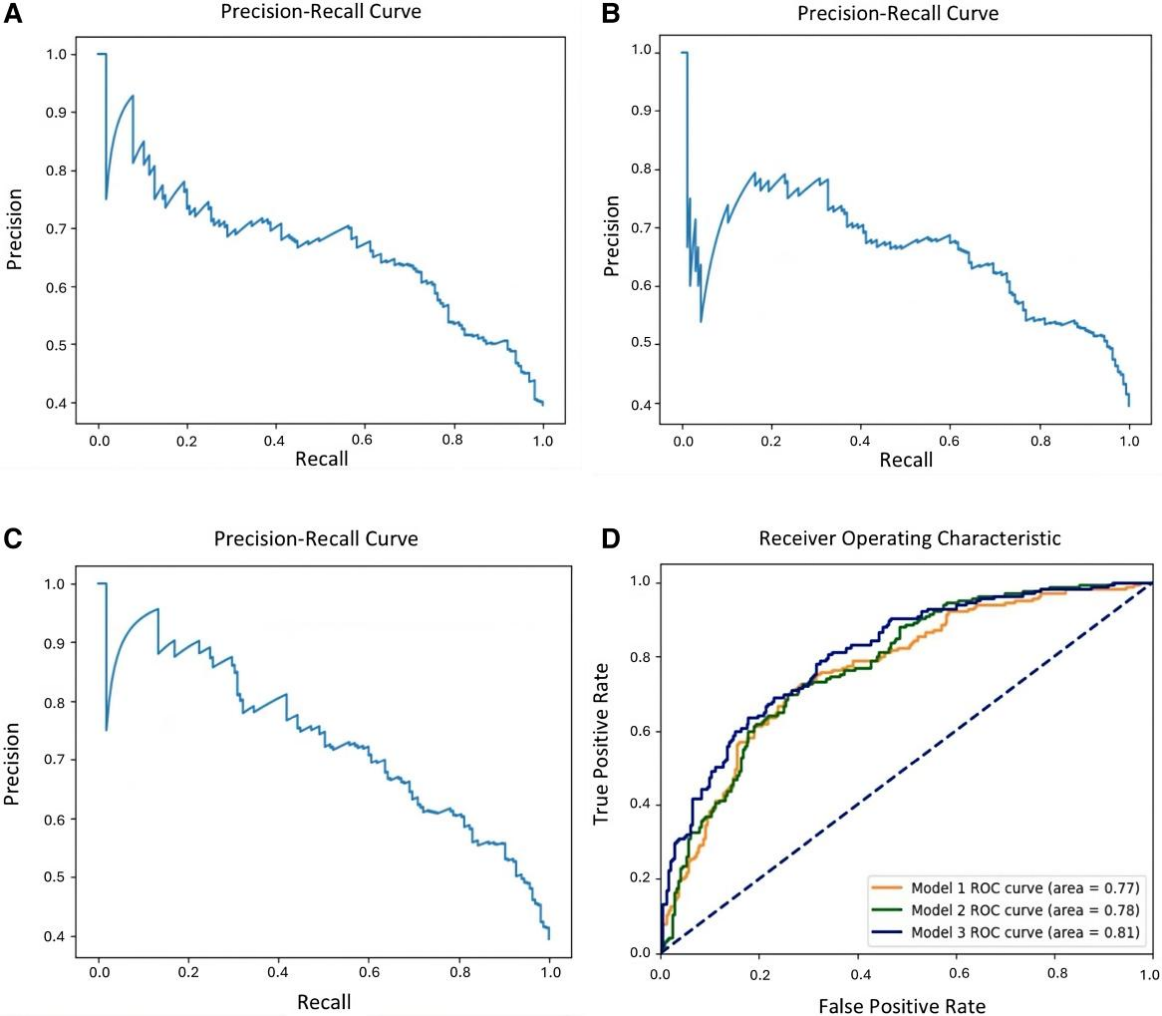
**Precision–recall curves and AUROC for label trials.** (**A–C**) Precision–recall curves for Models 1, 2 and 3, respectively. Model 1 trained and tested on only metadata, Model 2 trained on metadata and concept embeddings, and Model 3 trained on the aforementioned including abstract embeddings for prediction of label clinical trials. The models are plotted against their respective precision and recall outputs. Further shown are the ROC curves (**D**). Each model seen here is trained and tested for the years 1990–2017 with a random data split of 75:25 for training and testing, respectively.

Model 1 resulted with an AUC of 0.77, accuracy of 73.44%, precision of 70.15%, recall of 56.97%, F1 of 62.88% and lift score of 12.92%. This model achieved the lowest metric output out of each model for both translational outcomes. A full layout of the metrics for Model 1 label clinical trials is summarized in Table 4, and precision–recall curve is shown in Fig. 2A.

**Table 4:** Model 1: Metadata model label trials metric values and confusion matrix

Metric	Values (%)		Confusion matrix	
Accuracy	73.44		Predicted (0)	Predicted (1)
Precision	70.15	True (0)	213	40
Recall	56.97			
F1	62.88	True (1)	71	94
Lift	12.92			

Model 2 resulted with an AUC score of 0.78, accuracy of 73.68%, precision of 69.50%, recall of 59.39%, F1 of 64.05% and lift score of 13.16%. Model 2 for clinical trials performed similarly to Model 1; however, it has a slightly higher accuracy and lift score, full metrics and confusion matrix in Table 5. The precision–recall curve is shown in Fig. 2B.

**Table 5:** Model 2: Metadata and concepts model label trials metric values and confusion matrix

Metric	Values (%)		Confusion matrix	
Accuracy	73.68		Predicted (0)	Predicted (1)
Precision	69.50	True (0)	210	43
Recall	59.39			
F1	64.05	True (1)	67	98
Lift	13.16			

Our final model outperformed the previous two models for label clinical trials and was trained and tested on a combination of metadata, concept embeddings and abstract embeddings as feature input. This model outputted an AUC score of 0.81, accuracy of 75.11%, precision of 72.26%, recall of 60.00%, F1 of 65.56% and lift score of 14.59%. The model metrics are shown in Table 6, and the precision–recall curve is shown Fig. 2C.

**Table 6:** Model 3: Metadata, concepts and abstracts model label trials metric values and confusion matrix

Metric	Values (%)		Confusion matrix	
Accuracy	75.11		Predicted (0)	Predicted (1)
Precision	72.26	True (0)	215	38
Recall	60.00			
F1	65.56	True (1)	66	99
Lift	14.59			

To better visualize how each model for label trials performed, we plotted each AUC score onto one ROC curve (Fig. 2D). The ROC curve in this case depicts how each model performed compared with one another, the highest performing model being the final model with metadata, concept and abstract embeddings as feature input and the lowest performing model with the lowest accuracy and AUC being the initial model with only standard metadata as feature input.

### Application to other fields

In order to test the model’s applicability to other fields, we used the same methodology applied to cancer research papers. We experimented on the prediction label for patent inclusion with publications for breast cancer published between the years 2010 and 2015. We included standard metadata, concepts and abstract embeddings as feature input, down-sampling the majority label by 85%. The model resulted with an AUC of 0.81, accuracy of 75.27%, precision of 71.54%, recall of 89.44%, F1 of 75.73% and lift score of 23.24%. The confusion matrix and full metric outputs are summarized in Supplementary Table 4, and the ROC curve and precision–recall curve for the breast cancer model are shown in Supplementary Fig. 4.

## Discussion

This study represents, to our knowledge, the first study to predict the translational potential of dementia research using ML. Utilizing 43 091 publications from Dimensions, our ML model provides a novel approach, highlighting research papers that will lead to a patent or clinical trial inclusion. This work addresses a need, where the translation of fundamental research into practical applications has been notably slow, despite the substantial societal and economic burden of the disease.

It is within this context that we see the most promise for models that can help aid in the decision-making process. Within our data pool, only 8.99% of the data pool were cited in a patent or clinical trial. We present a model that can accurately predict whether a paper will be cited by a patent 77.17% of the time and can identify 80.72% of the papers that will go on to be cited by patents. We believe funders, research organizations and even scientific publishers could benefit from incorporating tools like this one into their decision-making processes.

### Model interpretation

The classification models we created can be used in conjunction with current translational research methods, such as the FAIT method. By integrating our ML model with these methods, we can uncover overlooked publications and expedite the identification of promising research. This synergy is crucial for advancing the field of dementia research, where the slow pace of progress and high failure rates in clinical trials have been long standing challenges.

The application of ML in this context is particularly promising, as it enables the handling of vast and complex data sets, a task impractical for traditional methods, simply by categorization. Our approach, using classification models, demonstrates the capacity of ML models to unravel intricate patterns and associations within academic research that has the capability to predict real-world impact. With large, unbalanced data sets, with preference towards negative labels, it is crucial to work with models that are effective and can achieve high accuracy levels. Most importantly, they are very suitable for real-time predictions, essential in the field of translational research where predicting a paper’s potential for further applications can speed up the process from basic research to clinical practice.

In developing our model, we drew inspiration from pioneering works in the field: Manjunath *et al.*^7^ patent correlation with research articles informed our decision to use patent citations as a measure of a paper’s translational potential. Nelson *et al.*^8^ analysed extensive databases using deep learning methods and demonstrated the superiority of advanced ML models over traditional citation metrics in predicting a research paper’s inclusion in patents and policies. We incorporated similar techniques, emphasizing the role of abstract embeddings in predicting research impact. Cao *et al.*^9^ further leveraged text mining and predictive modelling, exploring the transition of scientific concepts from basic research to practical applications, influencing our analysis of the content and context of research papers, specifically the inclusion of concepts and themes identified in publications. Finally, Li *et al*. introduced a novel TP measure, tracking the trajectory of biomedical research along the translational continuum.^10^ Our methodology was inspired by their approach, using biomedical knowledge representation to identify high-potential research.

### Model performance

We show that through analysis of our metadata, prediction outputs are inferior to those attained via high-dimensional vectors. Our analysis shows that the inclusion of concept and abstract embeddings, depicting overarching themes and trends within the publication, substantially improves the ML models prediction accuracy, extracting important information about themes necessary to classify publications.

Importantly, the ML model is capable of predicting whether newly published papers have potential for inclusion in patent or clinical trials. This signifies that through the correct frameworks it is feasible to execute high fidelity outputs that provide the field with a succinct path for translational output.

Contrary to traditional approaches that heavily rely on citation metrics, our findings suggest that these are not the sole indicators of a publication’s impact. Our ML model effectively identifies research likely to lead to patent or clinical trial citations without depending on time-sensitive data such as altmetric scores or the number of citations. This finding is pivotal in redefining the metrics used for assessing the translational potential of research.

We now have the option to incorporate ML models, with the capability to analyse complex thematic-based data, with our quantitative metrics, enabling us to identify themes and trajectories with the highest translational potential. We can use a supervised learning method, such as a classification model, alongside dimensionality reduction methods that capture large texts, translating from a high-dimensional vector to a low-dimensional space, enriching our data analyses. On the other hand, there has been opposing research stating that embeddings may be effective in shorter texts. Model performance has been seen to increase with abstract embeddings, which challenges the notion that whole-text embeddings may be superior. However, the exploration of whole-text embeddings versus abstract embedding only was outside the scope of our research.

### Limitations

While our study marks significant progress, it is not without limitations. The largest challenge lies with the nature of the publications and their requirement of time for patent or clinical trial inclusions. This means that it takes time for citations to be produced. To prevent time from being a large limiting factor, we removed time-sensitive features that could also leak the label to the model. This, in turn, allows for the application of the model to newly published papers and removes time as an obstacle in the model’s prediction.

The secondary challenge lies in the reliance on citations in patents and clinical trials as proxies for translational impact. This approach, while practical, may not fully capture the multifaceted nature of research impact. However, based on research by Manjunath *et al*., papers cited by patents seem to have distinct characteristics that are indicative of real-world impact. Based on the model’s increasing performance when including thematic-based features, we believe that our model is extracting intricate characteristics for patent and clinical trial prediction.^7^ We believe that the model’s increased predictive analysis, with features such as concept and abstract embeddings, provides a deeper understanding of otherwise-impossible-to-understand information.^8^ Even so, patent-cited articles are identified as a unique subset of biomedical literature, characterized by differences in scientific domain, research team composition and language use. This uniqueness poses a challenge in generalizing findings across different subsets of literature. This constraint, however, is amended by the model’s applicability to different domains, seen by its application to breast cancer research, alongside research presented by Cao *et al*.^9^ and Li *et al*.^10^

ML models are complex, making them both powerful in identifying patterns and challenging to simplify for understanding their key features. Despite the difficulty in explaining their intricacies, their complexity is essential for accurately analysing complex research, outperforming simpler models. This complexity versus simplicity issue highlights the trade-off between a model’s effectiveness and its explainability in translational research.

Our model’s feature weighting is not entirely transparent, and it might be subject to biases. Biases may be the result of multiple factors, such as unbalanced data for certain classes of papers, or under-representation of certain sub-populations in scientific literature. While there are several methods to test models against specific biases, this test was beyond the scope of the current research. At the same time, it should be noted that our model goes beyond simple citation counts used by current methodologies, analysing content and themes, potentially offering a more balanced view of a paper’s merit.

## Supplementary Material

fcae230_Supplementary_Data

## Data Availability

Data are accessible through Dimensions AI via an API login key. All code can be accessed via the following link: https://github.com/MatildaBeinat/ML-for-Translational-Research

## References

[1] Alzheimer’s Research UK . Prevalence and Incidence of Dementia. Accessed 15 December 2023. https://dementiastatistics.org/about-dementia/prevalence-and-incidence/

[2] Dimensions Research Integrity . Accessed 9 November 2023. https://www.dimensions.ai/products/research-integrity/

[3] Lemm D, von Rudorff GF, von Lilienfeld OA. Improved decision making with similarity based machine learning: Applications in chemistry. Mach Learn Sci Technol. 2023;4:045043.

[4] Hardy JA, Higgins GA. Alzheimer's disease: The amyloid cascade hypothesis. Science. 1992;256(5054):184–185. 1566067 10.1126/science.1566067

[5] Bassett DS, Gazzaniga MS. Understanding complexity in the human brain. Trends Cogn Sci. 2011;15(5):200–209. 21497128 10.1016/j.tics.2011.03.006 PMC3170818

[6] Si K, Li Y, Ma C, Guo F. Affiliation bias in peer review and the gender gap. Res Policy. 2023;52(7):104797.

[7] Manjunath A, Li H, Song S, et al Comprehensive analysis of 2.4 million patent-to-research citations maps the biomedical innovation and translation landscape. Nat Biotechnol. 2021;39(6):678–683. 34113042 10.1038/s41587-021-00940-5

[8] Nelson AP, Gray RJ, Ruffle JK, et al Deep forecasting of translational impact in medical research. Patterns (N Y). 2022;3(5):100483. 35607619 10.1016/j.patter.2022.100483 PMC9122964

[9] Cao H, Cheng M, Cen Z, McFarland DA, Ren X. Will this idea spread beyond academia? understanding knowledge transfer of scientific concepts across text corpora. Findings of the Association for Computational Linguistics: EMNLP 2020 [Internet]. 2020; Available from: 10.18653/v1/2020.findings-emnlp.158

[10] Li X, Tang X, Lu W. Tracking biomedical articles along the translational continuum: A measure based on biomedical knowledge representation. Scientometrics. 2023;128(2):1295–1319. 36570779 10.1007/s11192-022-04607-z PMC9758472

