# Supplementary material for: Machine learning to promote translational research: predicting patent and clinical trial inclusion in dementia research
Source: Brain Commun. 2024 Jul 25;6(4):fcae230. doi: 10.1093/braincomms/fcae230 (PMC11269431; doi:10.1093/braincomms/fcae230)
Supplement: fcae230_Supplementary_Data [file fcae230_supplementary_data.pdf]

Supplementary data

Supplementary Table 1

		Features included for label patents and clinical trials		
**Feature**	**Categorical Yes/No**	**Model 1**	**Model 2**	**Model 3**
Category rcdc (research, condition, and disease categorization)	Y	✓	✓	✓
Category hra (health research authority)	Y	✓	✓	✓
Category hrcs (health research classification system) rac (research activity code)	Y	✓	✓	✓
Reference ids count	N	✓	✓	✓
Recent citations	N			
Altmetric	N			
Relative citation ratio	N			
Times cited	N			
First author id	N	✓	✓	✓
First author name	Y	✓	✓	✓
First author affiliation id	N	✓	✓	✓
First author affiliation country	Y	✓	✓	✓
First author affiliation name	Y	✓	✓	✓
Funder countries	Y	✓	✓	✓
Authors count	N	✓	✓	✓
Funders	Y	✓	✓	✓
Journal id	N	✓	✓	✓
Journal title	Y	✓	✓	✓
Open access	Y	✓	✓	✓

Research organisation country names count	N	✓	✓	✓
Research organisation names	Y	✓	✓	✓
Research organisation names count	N	✓	✓	✓
Research organisation country names	Y	✓	✓	✓
Concepts	Y		✓	✓
Abstracts	Y			✓

Full feature list with metadata that is found within the Dimensions database, alongside features included in each model. The definition of each feature can be found in the supplementary data (Supplementary Table 2).

Supplementary Table 2

Feature	Definition
Category hra	Categorised by health research areas.
Category hrcs rac	Categorised by health research classification system and their research activity codes.
Category rcdc	Categorised by research, condition, and disease.
Reference ids	There are IDs for publications in the reference list of the original publication.
Recent citations (excluded from feature list)	The number of citations the publication received in the last 2 years.
Altmetric (excluded from feature list)	The attention score based on the weight count of attention received by the publication across the internet.
Relative citation ratio (excluded from feature list)	Citation performance of the paper relative to other papers in the same field.
Times cited (excluded from feature list)	The number of times the publication has been cited.
First author id	The ID of the first author that created the publication
First author name	The name of the first author that created the publication.
First author affiliation id	The affiliation ID of the first author in the publication
First author affiliation country	The affiliated country of the first author in the publication

First author affiliation name	The name of the affiliation of the first author in the publication
Authors count	The number of authors in the publication.
Funder countries	The country of the organisations that fund the publication.
Funders	Organisation funding the publication.
Journal id	The journal that the publication belongs to.
Journal title	The title of the journal that the publication belongs to
Open access	Publications category determining whether it is free access: Gold (free), Bronze (available on publisher's website), Hybrid (available under open licence), Green (available in open access repository), Closed (not free).
Research organisation names	The names of the organisations the authors are associated with.
Research organisation names count	The number of research organisations related to the authors in the publication
Research organisation country names	The country names of the organisations the authors are associated with.
Concepts	Keywords in the publication.
Abstracts	Entire abstract of individual publications

Feature definitions of features considered for the ML models.

Supplementary Figure 1. Workflow diagram of publications.

Workflow description to achieve final publications for both patents and clinical trials.

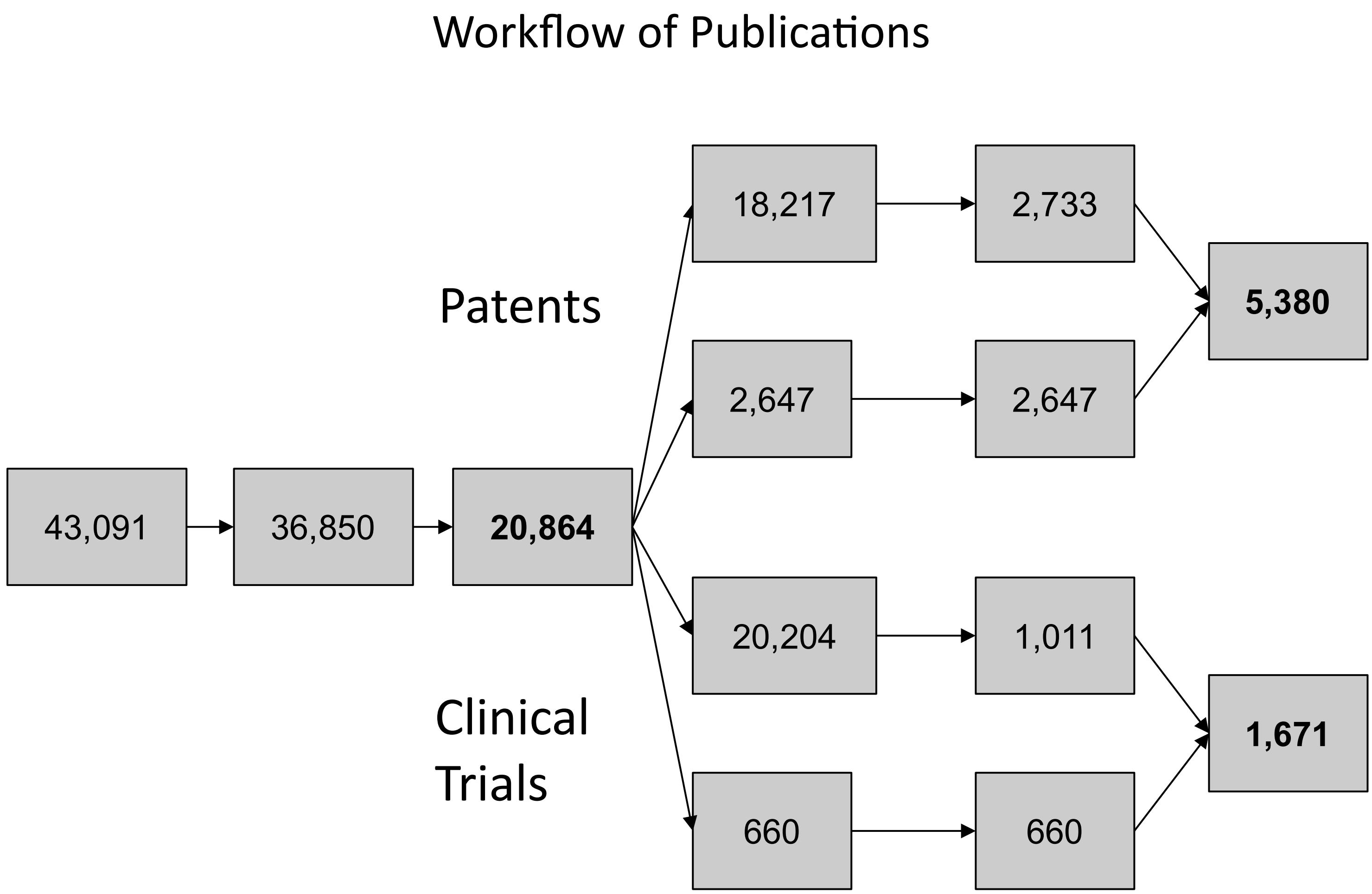

Supplementary Figure 2. Time delay from paper publication to initial patent citation.

The average time delay from paper publication to initial patent citation is of 4.28 years, with a standard deviation of 4.57.

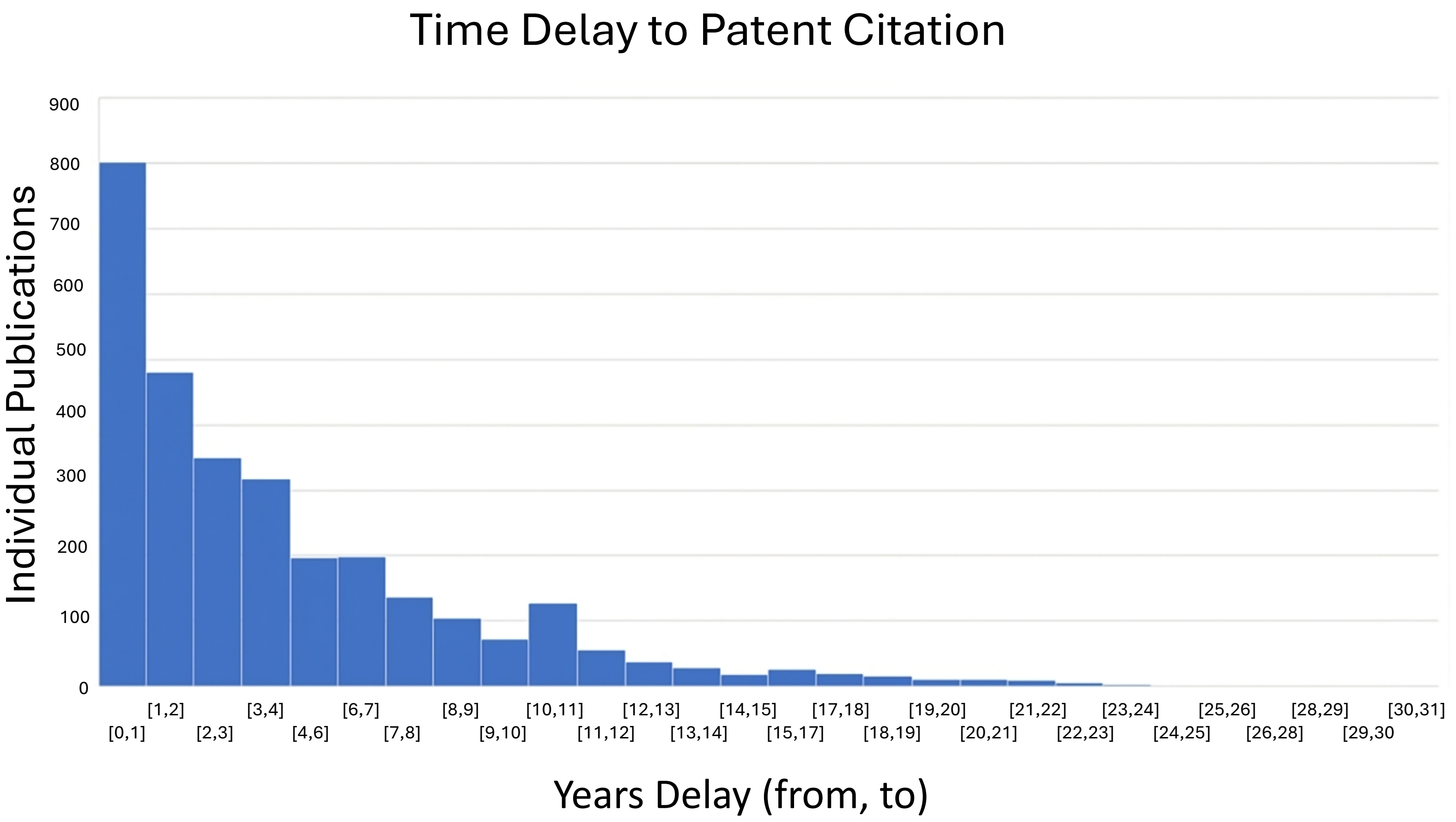

Supplementary Figure 3. Time delay from paper publication to initial clinical trial citation.

The average time delay from paper publication to clinical trial citation was 6.57 years, standard deviation of 5.77.

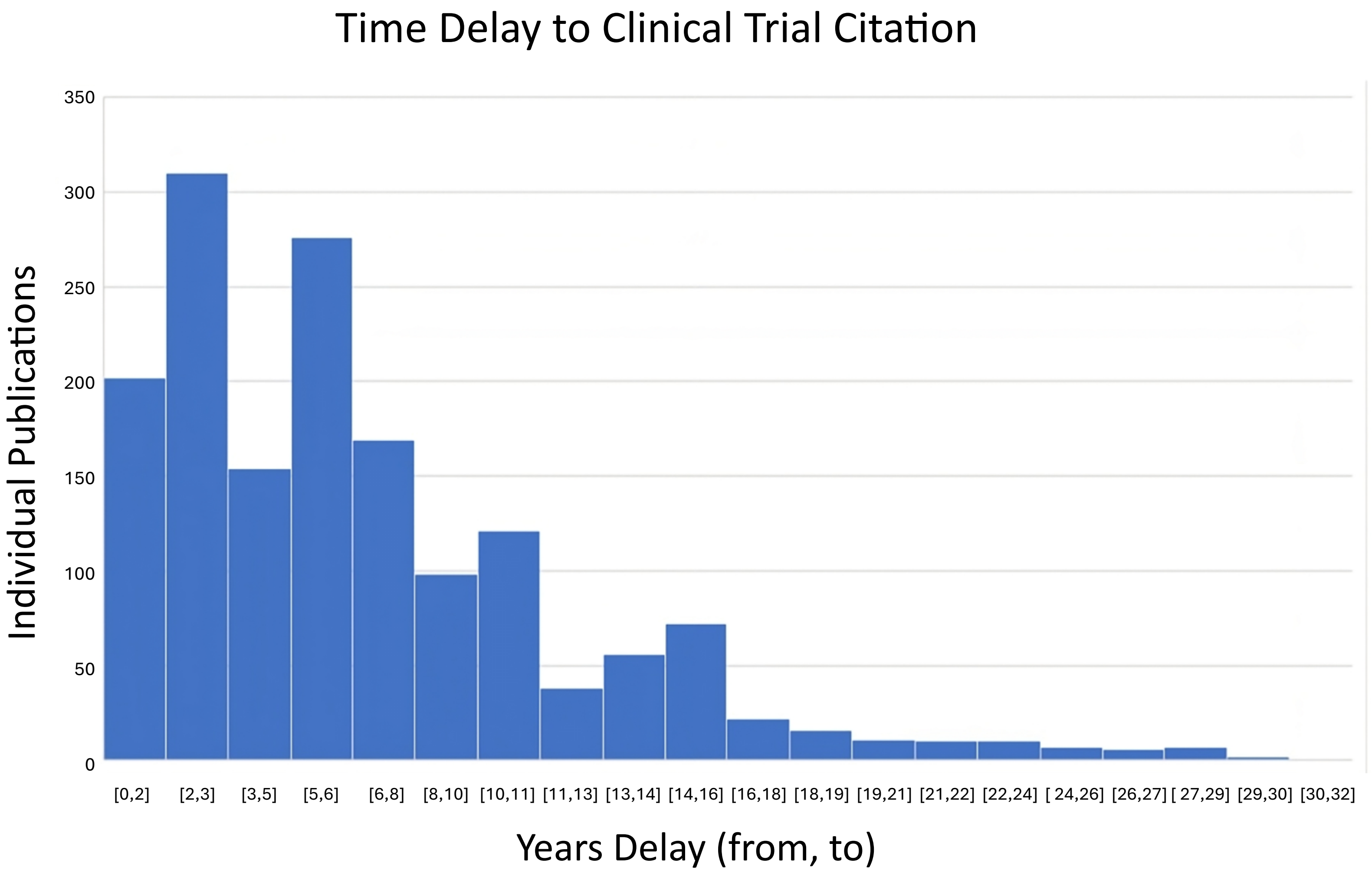

Supplementary Table 3

			Predicted label	
			0	1
**Real label**	**1990-2000**	**0**	38	12
		**1**	12	36
	**2000-2010**	**0**	36	14
		**1**	13	37
	**2010-2017**	**0**	32	8
		**1**	9	31

Confusion matrices for each decade in the predicted label patents (Figure 1E).

Supplementary Table 4

Metric	Values	Confusion matrix		
Accuracy	75.27%		Predicted (0)	Predicted (1)
Precision	71.54%	True (0)	172	72
Recall	80.44%			
F1	75.73%	True (1)	44	181
Lift	23.24%			

Breast cancer experiment model for label patents. Metric values and confusion matrix.

Supplementary Figure 4. ROC and precision-recall curves for breast cancer experiment model label patents.

Shown is the ROC curve (A) where the yellow curve depicts the model trained on standard metadata, concept and abstract embeddings as feature input. The model is trained and tested for the years 2010-2015 with a random data split of 75:25 for training and testing respectively. (B) Precision-recall curves for the breast cancer experiment model plotted against the respective precision and recall outputs.

A

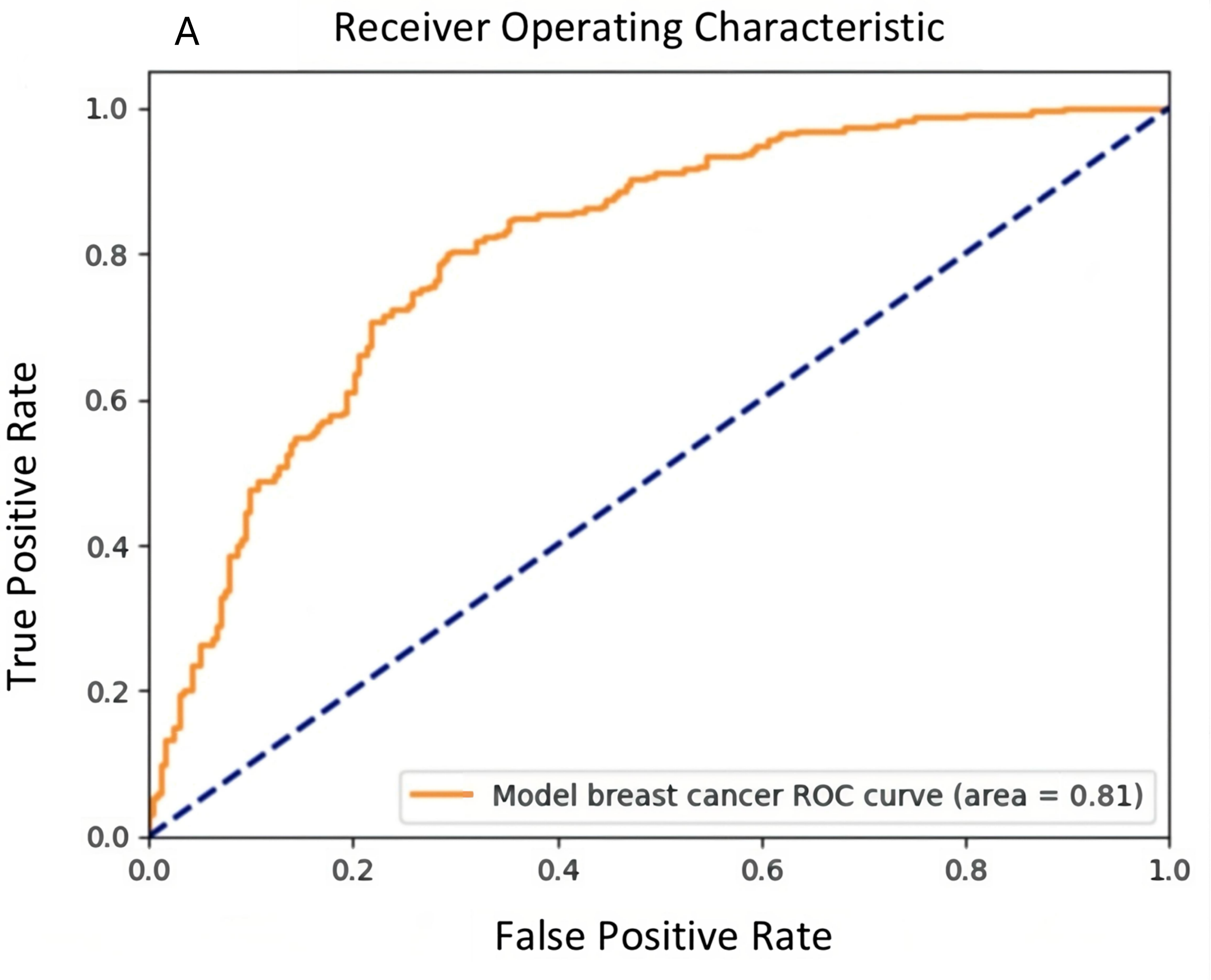

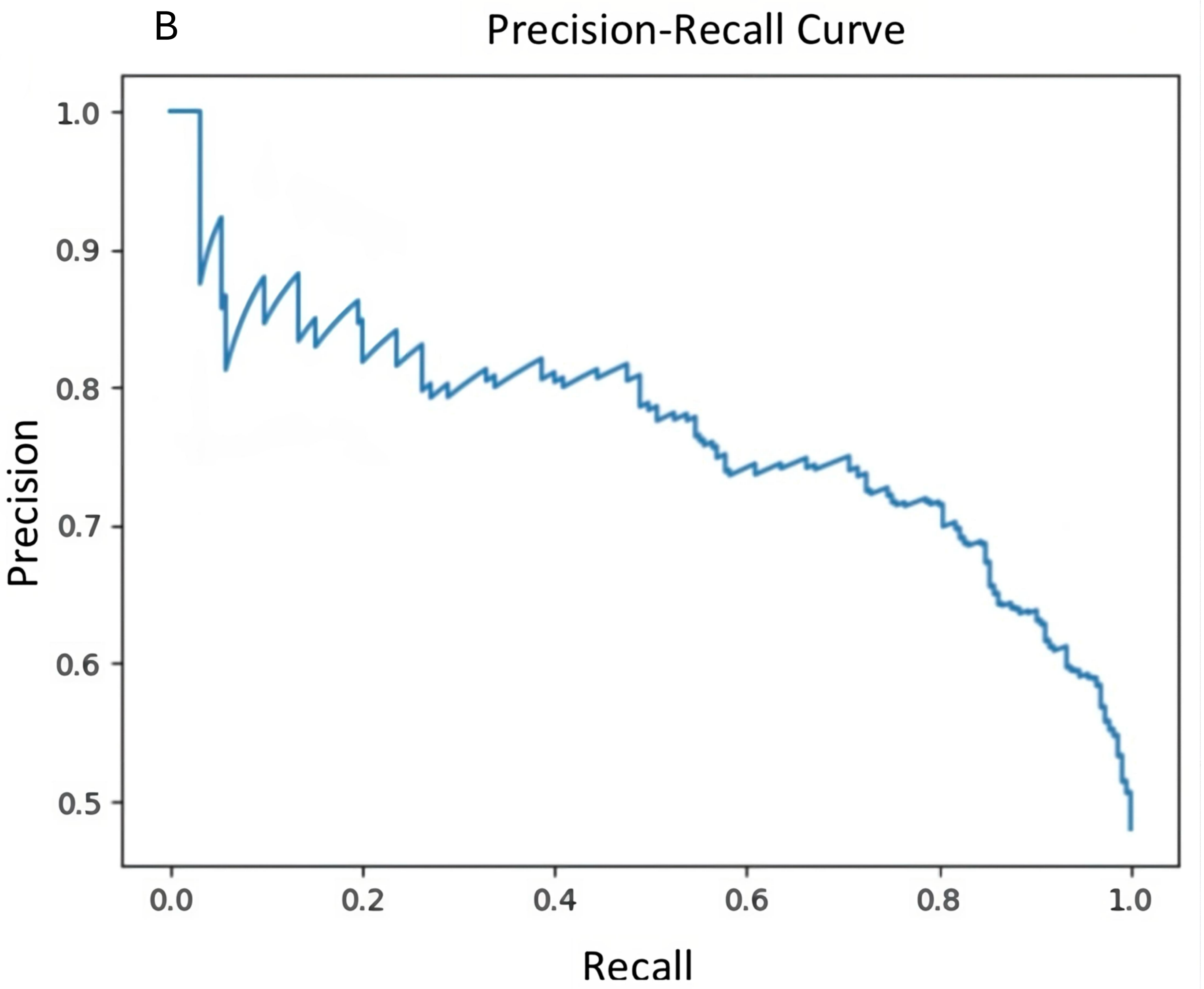
B
Precision-Recall Curve
Precision
1.0
0.9
0.8
0.7
0.6
0.5
0.0
0.2
0.4
0.6
0.8
1.0
Recall

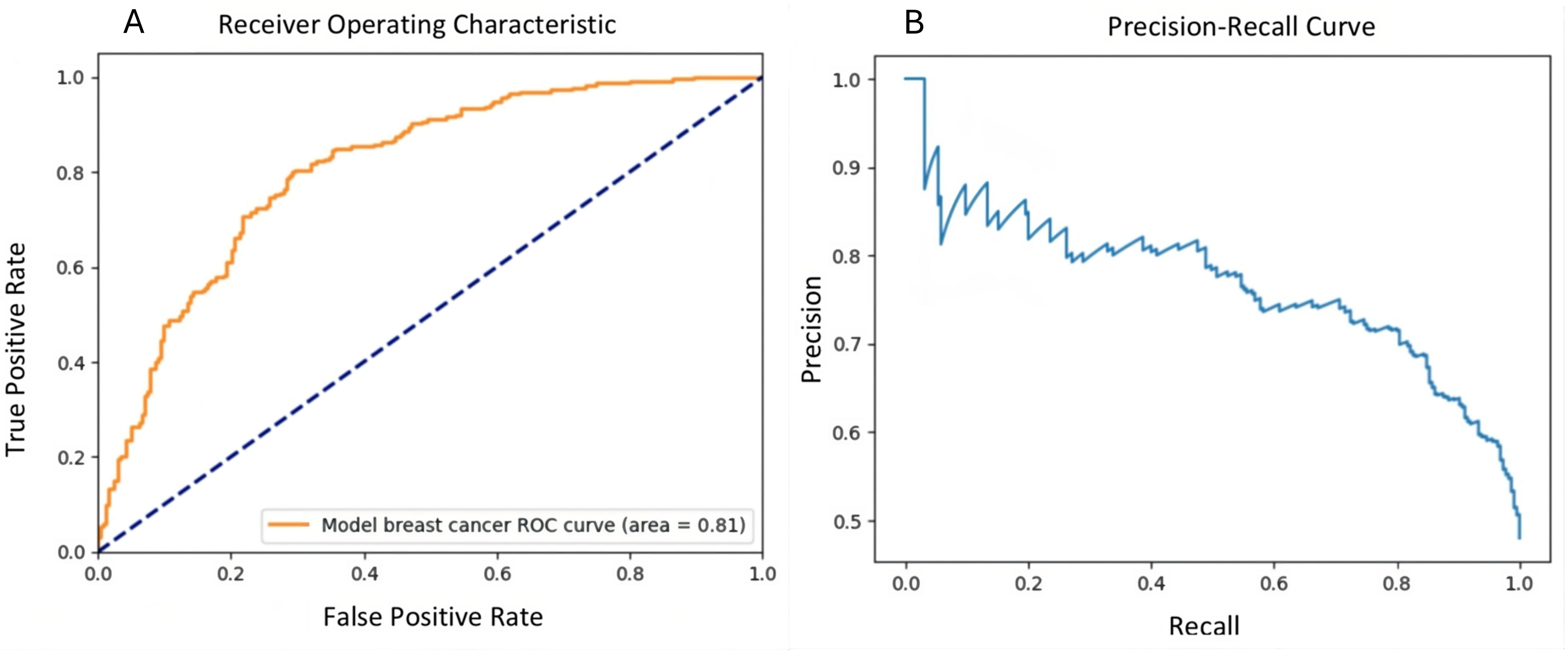
A
Receiver Operating Characteristic
True Positive Rate
False Positive Rate
0.0
0.2
0.4
0.6
0.8
1.0
Model breast cancer ROC curve (area = 0.81)
B
Precision-Recall Curve
Precision
Recall
0.5
0.6
0.7
0.8
0.9
1.0
0.0
0.2
0.4
0.6
0.8
1.0
